# Identification of syncytin-car-1, an endogenous retroviral envelope gene involved in placentation and conserved in Carnivora: a syncytin in a new superorder of placental mammals

**DOI:** 10.1186/1742-4690-8-S2-P13

**Published:** 2011-10-03

**Authors:** Guillaume Cornells, Odile Heidmann, Sybille Bernard-Stoecklin, Géraldine Véron, Karine Reynaud, Baptiste Mulot, Anne Dupressoír, Thierry Heidmann

**Affiliations:** 1 CNRS UMR 8122, Institut Gustave Roussy, Villejuif 94805, France and Université Paris-Sud, 91405 Orsay, France; 2 Systématique et Evolution, CNRS UMR7205, Muséum National d'Histoire Naturelle, Paris, 75231, France; 3 INRA-ENVA UMR 1198, Ecole Nationale Vétérinaíre d'Alfort, Maísons-Alfort, 94700, France; 4 Zoo Parc de Beauval, St Aignan surcher, 41110, France

## Background

A large fraction of vertebrate genomes is composed of endogenous retroviruses (ERVs), which are remnants of ancient retroviral infections. In mammals, five retroviral envelope genes with a placenta-specific expression and a fusogenic activity, the syncytins, have been independently "captured" in primates, rodents and lagomorphs (which are all Euarchotonglires mammals) and conserved over more than 30 million years in a functional state. They are involved in the formation of the syncytiotrophoblast, and are necessary for placental development. Here we searched for syncytins in the Carnivora order that belongs to the Laurasiatheria, which diverged from the Euarchotonglires shortly after mammalian radiation around 100 million years ago.

## Methods

Dog and cat genomes were screened for full-length retroviral envelope genes. Candidate genes were assayed for their placenta-specific expression by quantitative RT-PCR of RNAs isolated from a large set of tissues, and by *in situ* hybridization on placental tissue sections. Phylogenetic conservation was investigated by cloning and sequencing of the candidate genes in species representative of major Carnivora branches. Fusogenic activity was assayed by an *ex vivo* infectivity assay with viral pseudotypes.

## Results

4 and 7 retroviral envelope gene families were found in the dog and cat respectively. Among them, we identified an envelope gene shared by both species and specifically expressed in the placenta at the materno-fetal barrier, where syncytiotrophoblasts can be found. It is conserved at the same genomic locus in the 25 species tested, presents strong signature for purifying selection, as usually observed for a host cellular gene, and has conserved its fusogenic properties in all species. This gene presents all the characteristics of a *bona fide* syncytin gene.

## Conclusion

We identified a sixth syncytin in the Carnivora order, syncytin-Car-1. It is the oldest syncytin identified to date, conserved for at least 70 million years, and the first one identified in the Laurasiatheria. Identification of this new syncytin in a major branch of eutherian mammals shows that syncytin gene capture is a very general process, which occurred in several branches during eutherian evolution. The carnivora placental structure is slightly different from that of primates, rodents and lagomorphs (endotheliochorial vs haemochorial), and characterization of the syncytins found in various species might help to understand the impact of these independently acquired elements on placental diversity. Study of syncytins brings new insights on the evolutionary relationship between ERVs and their host.

